# An MRI template of the female C57Bl/6N mouse brain

**DOI:** 10.1080/07853890.2026.2753239

**Published:** 2026-11-11

**Authors:** Peter Stöhrmann, Orsolya Horvath, Joachim Friske, Thomas Wanek, Thomas H. Helbich, Andreas Hahn, Rupert Lanzenberger, Daniela D. Pollak

**Affiliations:** ^a^ Division of General Psychiatry, Department of Psychiatry and Psychotherapy, Medical University of Vienna, Vienna, Austria; ^b^ Comprehensive Center for Clinical Neurosciences and Mental Health (C3NMH), Medical University of Vienna, Vienna, Austria; ^c^ Division of Neurophysiology and Neuropharmacology, Center for Physiology und Pharmacology, Medical University of Vienna, Vienna, Austria; ^d^ Division of Molecular and Structural Preclinical Imaging, Department of Biomedical Imaging and Image-guided Therapy, Medical University of Vienna, Vienna, Austria

**Keywords:** Brain, template, atlas, MRI, mouse, female brain

## Abstract

**Background:**

Brain templates are pivotal tools for standardized image analysis and interpretation in neuroimaging studies. However, matching data and reference remains essential. With growing awareness of sex as a relevant biological variable and male-centric biases in past medical investigations, the continuous limited use and availability of female-specific brain templates, particularly in preclinical research are alarming.

**Methods:**

To address this gap in preclinical neuroimaging, we created a high-resolution, *in vivo* structural MRI brain template for female C57Bl/6N mice, a population-average derived from 153 axial and coronal scans of 80 female mice. We further analyzed the template’s characteristics regarding the achieved effective resolution and compared it to currently available standard brains. Validation was performed by spatially normalizing external data to established templates and the novel reference, contrasting the degree of deformation.

**Results:**

We present a readily applicable and accessible set of files (structural images, tissue probability maps, anatomical annotations, and transformation matrices) as reference for female-centric neuroimaging research questions. The novel template brain exhibits high nominal and effective resolution, differs significantly in spatial and cohort characteristics from previous references and shows improved performance with external data of female mice.

**Conclusion:**

We propose that integrating sex-specific tools into preclinical neuroimaging studies is essential for reducing sex bias, enhancing data reproducibility and strengthening the translational validity of neuroscientific findings. Research further disentangling (sub-)strain- and sex-differences is warranted.

## 1. Introduction

Brain templates and atlases are essential tools in neuroscience research [1]. They serve as references for standardized image processing, translation, interpretation, and reporting of experimental data. Specific resources for different species and developmental stages are currently available, that represent various dimensions of structural, functional, or genetic modalities of the brain. Despite their vital role in advancing our understanding of the nervous system, the applicability and limitations of these resources should be carefully considered when working with or referring to a specific brain atlas or template [2]. The underlying (imaging) modality and methodology, but more importantly, the oftentimes very specific and restricted input data set needs to be taken into account [3,4].

Tailored tools for certain age groups [5–10], nationalities and/or ethnicities [11–14] and pathologies [15,16] are available for the human brain and deemed essential [17], to avoid biases from the application of ill-matched references [7]. However, specific templates appear to be used to a lesser extent to cover sex-specific aspects, particularly in preclinical research. The limited number of previous works were either not widely disseminated or are not openly accessible [3,11,18]. However, differences between sexes^1^ in total brain volume, structure, function and temporal development have been widely reported [3,18–24]. Models have even been applied to classify human brains by sex based on anatomical characteristics (e.g. [25,26], but discussed critically in [27,28]). Recently, dimorphism in the Drosophila central nervous system’s connectome has also been reported [29].

A by no means exhaustive selection of templates, aiming to provide a gross overview of human and mouse (one of the most common preclinical models in neuroscience research [30]) neuroimaging templates and their underlying data characteristics can be found in Table 1. A more in-depth review of available mouse atlases, also highlighting the aforementioned paucity of and need for female data-sets in *in vivo* mouse studies, has been provided elsewhere [4,31]. To the best of our knowledge, there are currently only two annotated female templates openly available [31,32], one of which is derived from the most studied C57Bl/6 strain, but it exhibits low, highly anisotropic resolution across slices. Physiological, morphological and potentially related behavioral differences between various inbred [33,34] and outbred mouse strains, including J and N C57Bl/6-substrains [36] (see also Table 2), may further limit their broad applicability.[35]

**Table 1.** Compilation of neuroimaging templates and their experimental characteristics.

Template (human)		Modality	N	n	Age [y]	Resolution [mm]	Comment
MNI-ICBM [37–39] □		MRI T1	152	66	18–44	0.74 isotropic	Various versions, included in SPM, MRIcroGL
**Template** **(mouse)**	**Strain**	**Modality**	**N**	**n**	**Age**	**Resolution[µm]**	**Comment**
Duke Mouse Atlas (DMA) [34] □	C57Bl/6**J** DBA/2J CAST/EiJ BTBR	MRI DWI *ex vivo*	8 8 8 8	4 4 4 4	∼90 d	45 isotropic	Requires online registration^2^, access was approved within 24h [*] Followed up by multi-scalar atlas [40] **Sex-specific averages** also available
Allen Mouse Brain Atlas (AMBA) [41] □	C57Bl/6**J**	Microscopy, atlas	?	0	?	0.95 × 0.95 × 25	Serial two photon tomography in NIfTI format available^3^
Allen Mouse Brain Common Coordinate Framework (CCFv3) [42]	C57BL/6**J**	Two-photon tomography	1 675	621		10 isotropic	Integration of MRI data by Wang et al. [43]
The Institute of High Energy Physics Mouse Template (IMT) [44]	C57Bl/6**J**	MRI T1 (MEMRI) MRI T2	15 23	0 0	10–11 w	60x60x500 60x50x200	
Mouse_M.Mirrione [45,46]	C57Bl/6**J**	MRI T2* *ex vivo*	10	0	12–14 w	47 isotropic	Included in PMOD
Paxinos and Franklin’s The mouse brain in stereotaxic coordinates [47]	C57Bl/6**J**	Atlas	?	0	?	Not applicable	Distance between sections varies
DSURQE [48–52] □	C57Bl/6**J**	MRI T2 *ex vivo*	40	20	12 w	40 isotropic	Included in RABIES [53]; retrieved from the Mouse Imaging Centre (MICe)^4^
Mortimer Space Atlas [20] □	C57Bl/6**J**	MRI T2	60	30	8–12 w	70 isotropic	Requires online registration^5^, approved immediately
Templates for *In vivo* Mouse Brain [33] □	C57Bl/6(?) BALB/cBy C3H/He DBA/2	MRI T1 *in vivo*	30 10 10 10	0 0 0 0	17 w	80 isotropic	Templates for four different strains available on NITRC
MouseX (female-only) [32] □	C57Bl/6**J**	MRI T2, DWI *in vivo*	30	30	56–60 d	70x70x400	Separate templates for females, males and both sexes combined
Turone Mouse Template and Atlas (TMBTA) [31] □	RjOrl:SWISS	MRI T2 *in vivo*	23 (?)	23	8 w	60 isotropic	Integration of AMBA and AMBMC [54] templates and annotations Available on NITRC

Female-only templates are marked in grey. Squares (□) denote MR templates accessible to the authors online at the time of writing without personal request. N is used to indicate the population size, n the number of female individuals included.

**Table 2.** Exemplary d*ifferences between* and characteristics of *C57Bl*/*6* J and N *sub-strains*. Of note, not all differences have been tested in either sex.

	C57Bl/6J	C57Bl/6N
Morphology	≈10 % incidence of congenital portosystemic shunt (PSS) increases brain weight and volume [71] variability in circle of Willis/posterior communicating artery [72]	*No similar or contrasting reports in C57Bl/6N to the best of our knowledge*
Physiology	more severe experimental autoimmune encephalomyelitis [73] higher proinflammatory profile [73] prolonged Propofol anesthesia [74] PSS alters neuro-metabolite profile [71] more sensitive to noise trauma [75] reduced ischemic consequences due to postnatal hypoxic-ischemic brain injury [76]	less expressed obesity phenotype [77] higher anti-inflammatory profile [73] distinct differences in vascular function [78] prolonged Midazolam anesthesia [79] reduced ketamine-withdrawal-induced psychotic-like symptoms [80] more sensitive to some effects of chronic corticosterone treatment [81] unsuitable for drug ototoxicity studies [75]
Behavior	higher baseline locomotor activity [58] enhanced motor coordination on rotarod apparatus [82,83] higher exploratory activity [83] higher active social engagement [83] reduced exploratory activity and heightened anxiety with age [84] PSS alters behavior, e.g. hyperlocomotion [71]	significantly higher freezing behavior [82] lower baseline pain sensitivity [82] aging reduces social novelty preference [84]
Genetics	spontaneous 5-exon deletion in the Nnt (nicotinamide nucleotide transhydrogenase) gene regulating mitochondrial energy regulation	spontaneous rd8 mutation in the Crb1 gene leads to retinal degeneration

To bridge the existing gap in the availability of sex-specific resources, we report the availability of a high-resolution, cohort-derived, template of the female mouse brain. It includes tissue probability maps, spatial transformations from other common standards and spatially matched annotations. The underlying data was collected within a multi-armed, longitudinal structural and functional magnetic resonance imaging (MRI) study in female C57Bl/6N mice.

### 1.1. Strain and sex differences in rodents

Despite their smooth surface and lack of gyrification, mouse brains may differ significantly between strains, both in structural and functional aspects. Genetical manipulations, different physiology, vulnerability to certain diseases and various behavioral phenotypes extend the spectrum.

Scholz et al. have previously shown that three common mouse strains (C57Bl/6J, CD-1, 129S6) significantly differ in brain surface anatomy, specifically in motor, anterior somatosensory, retrosplenial and entorhinal cortex. Already two principal components could be used to differentiate strains with 91% accuracy [35]. Skeletal, specifically skull measures were also found to be divergent between mouse strains [55] and sexes in rats [56]. Importantly, phenotypical differences are not only prevalent between strains, but even between sub-strains [57]. For the C57Bl/6 sub-strains C57Bl/6J and C57Bl/6N, substantial divergence in behavioral and neurological function has been reported [36,58–60]. Table 2 summarizes the most prominent differences in these two C57Bl/6 sub-strains. Even between 6J sub-lines, neuroanatomical and behavioral divergence has long been observed [61,62]. Against this background, there is a justified need to create currently lacking brain templates and atlases for the N-sub-strain.

Sexual dimorphism in murine brain structure and function has repeatedly been reported. Interestingly, studies in humans reporting males to have larger and more variable total brain volume, have not been reproduced in rodents [63]. However, neural differences at the cellular and synaptic levels (e.g [64,65].) have been observed, complementing macroscopic differences [66] and sex-dependent results in structural and diffusion-weighted magnetic resonance imaging [22,32,52,67,68]. These data strongly indicate specific brain areas including the (medial) amygdala, bed nuclei of the stria terminalis and hypothalamic subregions. In awake rats, resting-state functional MRI identified sex-specific network organization and connectivity strength [69]. Male and female mice on the other hand showed differences in BOLD activation to mechanical stimuli [70].

Hence, although in comparison to the human brain, the mouse brain might show less striking anatomical variability, known sexual dimorphisms may still bias nonlinear registration and atlas-based quantification of (f)MRI data. A brain template derived of scans of female and male brains constitutes an average that might not be optimally suited to represent either sex. Consequently, registering female datasets onto a mixed-sex template may result in local deformations and/or diminish sensitivity to the detection of female-specific neuroanatomical effects. It remains to be determined if and how sex-related registration bias may have impacted the results obtained in previous studies relying on the use of mixed-sex templates, but this is inherently depending on the availability of a sex-specific resource.

## 2. Materials and methods

### 2.1. Subjects

All experimental procedures were approved by the National Ethics Committee on Animal Care and Use (*Bundesministerium für Wissenschaft und Forschung*, GZ: 2021-0.150.413) and were conducted by trained personnel. ARRIVE guidelines were adhered to as applicable (see Supplemental Material).

Eighty female C57Bl/6N(Crl) mice were obtained from Charles River (Sulzfeld, Germany) in two cohorts of forty mice each. They were housed in pairs, in individually ventilated cages (*Tecniplast*, Italy), on a 12-hour dark-light cycle, with *ad libitum* access to water and standard chow (Breeding/Maintenance Diet - Rats/Mice, *Altromin Spezialfutter GmbH & Co. KG*, Germany). Cages were equipped with nesting material and a wooden gnawing stick. Upon arrival in the facility, the mice were allowed to habituate for a minimum of 13 days prior to the MR scan. A larger age span of 69–108 days at time of scanning was consciously chosen to robustly represent the ‘adult’ female brain. Covering a range of several weeks ensures wider applicability [85,86] and aligns with age ranges used in templates for healthy human adults [37–39].

### 2.2. *In vivo* MRI acquisition

Magnetic resonance images (MRI) were recorded at the facilities of the Preclinical Imaging Laboratories, Department of Biomedical Imaging and Image-guided Therapy, Medical University of Vienna. A 9.4 T μMRI scanner (BioSpec 94/30 USR, Bruker Germany) running ParaVision 360 v3.3 software, in combination with a mouse-brain-specific, transmit/receive, helium-cooled surface coil (*CryoProbe* Z125365) was used.

Anesthesia was induced by placing an animal in a chamber filled with 3.5% isoflurane in medical air. The mouse was positioned in a headfirst, prone position on a water-heated MRI bed. The head was fixed using a bite bar and net dressing, fastened at the rear end of the bite bar and 3D-printed anchors. Ear bars were not used. Eyes and ear canals were covered respectively with ointment (*Bepanthen^®^ Augen- und Nasensalbe*, Bayer Austria, containing 50 mg/g (+)-Panthenol) and cotton balls to reduce scanner noise.

The maintenance phase of anesthesia was initiated by an intramuscular injection of 0.1 mg/kg medetomidine hydrochloride (*Domitor^®^*, 1 mg/ml, *Orion Pharma*) in the hind legs. Subsequently, the isoflurane concentration was gradually reduced during the anatomical scans to 0.5% for later functional MRI acquisitions, for which combined anesthetic regimens such as isoflurane and medetomidine are currently regarded as state-of-the-art [87]. However, the analysis of and reports on the functional data are beyond the scope of this paper. While deep anesthesia may be favorable to limit movement during structural recordings, this sequence order (structural, followed by functional scans) allowed for gradual isoflurane reduction, without having to pause functional image acquisition for isoflurane effects to subside, hence reducing the total exposure to anesthesia. Due to aforementioned head fixation, motion artefacts were nonetheless negligible.

As overall acquisition time was less than one hour, medetomidine was only injected prior to and not infused continuously during scanning. Venous cannulation could therefore be omitted. Of note, due to physiological effects such as vasoconstriction, at a small scale and high resolution, both functional and also structural images might be affected differently by various anesthetic substances [88].

Upon positioning in the scanner and verification of placement and signal quality (matching and tuning), shimming, B0 field mapping, and two consequent anatomical scans per mouse were performed. To retrospectively confirm the absence of significant image distortions in the center of the scanner bore, a 3D-printed phantom featuring both a regular, orthogonal grid and the shape of a mouse skull was constructed and measured with both CT (i.e. inherently free of distortions) and MR (see Supp. Figure 1).

**Figure 1.**
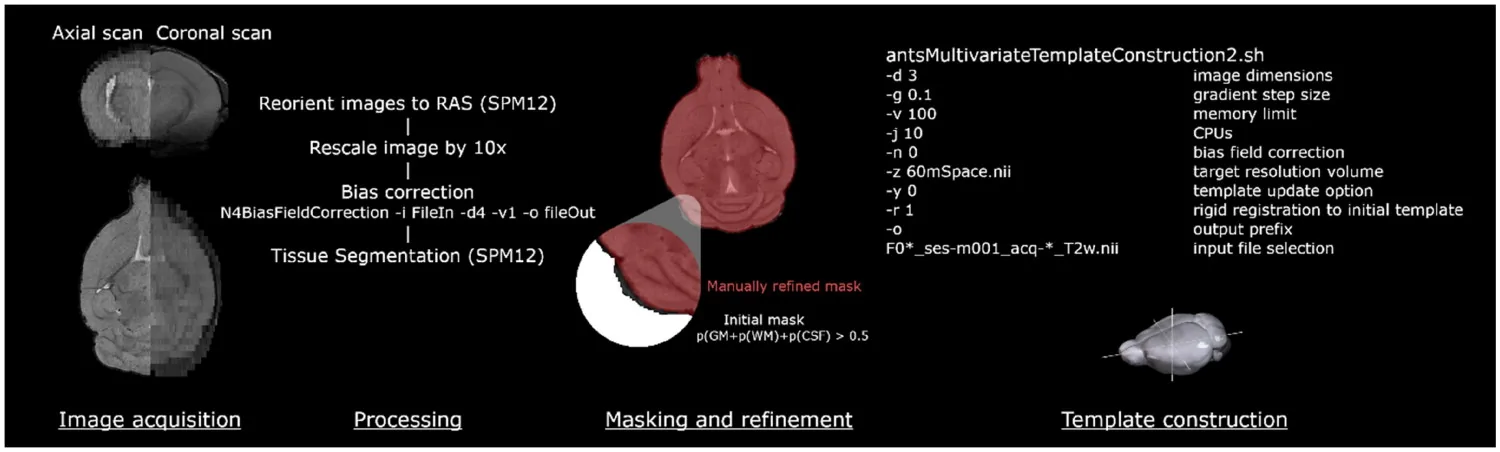
Schematic data acquisition and template creation process. Orthogonal, anisotropic scans of high in-plane but lower through-plane resolution (image acquisition, left) are processed and masked to construct an isotropic template.

The respiratory activity of the animals was constantly monitored during scans (SA Instruments, Inc., Stony Brook, NY, USA). The structural sequence parameters were T2 TurboRARE: **1)** TE/TR = 40/3200 ms, 3 averages, matrix 256 × 256, resolution 75 × 75 µm (axial), slice thickness 250 µm, scan duration 05:07 (excluding gating), and **2)** TE/TR = 40/3163 ms, no averaging, resolution 78 × 38 µm (coronal), slice thickness 500 µm, scan duration 01:41 (excluding gating). As respiratory gating was observed to introduce subtle, but nevertheless notable intensity inhomogeneities between planes of axial scans, and movement was generally low, gating was abandoned in the course of the study. This resulted in even shorter acquisition duration and total time under anesthesia (<45 min). Twelve minutes of resting-state BOLD fMRI (480 frames) data were also recorded.

### 2.3. *Ex vivo* MRI acquisition

A post-mortem scan of a single perfused brain was recorded for additional quality control steps: To rule out that combining anisotropic, axial and coronal scans during template creation introduces a volumetric bias, the image protocol was extended with a T1-weighted, 50 µm-isotropic sequence. Due to its much longer acquisition time (around 30-fold), this high-resolution scan was not implemented in the *in vivo* measurements.

### 2.4. Template creation

Structural scans were exported from ParaVision as Dicom and converted to NIfTI *via* dcm2niix [89] as implemented in MRIcroGL [90]. These files were spatially upscaled (10x), realigned, bias-corrected (*N4BiasFieldCorrection* with default values) and masked, using custom MATLAB scripts (R2023b, MathWorks), SPM12 (v7771) and tissue probability maps (TPM) of the TMBTA template for initial segmentation of brain tissue, by thresholding the sum image of individual TPMs. Manual mask refinement (i.e. additional inclusion or exclusion of voxels), as depicted in Figure 1, was performed in 3D Slicer (Slicer 5.6.2, www.slicer.org) [91]. Scans were visually inspected for image quality and homogeneity, leading to a final selection of 153/160 scans (76 axial, 77 coronal).

Advanced Normalization Tools [92] (ANTs, http://stnava.github.io/ANTs/, *antsMultivariateTemplateConstruction2.sh,* running on Debian GNU/Linux 11 (Bullseye)) was then used for template creation and spatial transformations (*antsApplyTransforms*, *antsRegistrationSyN.sh*). The resulting **Fe**male **Bl**ack **S**ix template brain (FeBls, *‘Fabulous’*) was then masked once more for any remaining non-brain tissue.

To achieve a 60 µm isotropic, high-SNR structural template, we combined axial and coronal scans using ANT’s multivariate template construction (*antsMultivariateTemplateConstruction2*). A schematic with representative images and code of the described procedure are shown in Figure 1.

To exclude the possibility of introducing a volumetric bias by this procedure, a perfused brain was not only scanned at the same anisotropic resolutions previously described, but also at 50 µm isotropic. Each of these three scans, as well as a second isotropic image, derived from the anisotropic recordings by applying the approach described above, was then normalized to the template space and parcellated. Consequently, the segmented volumes in the individual space were compared to the high-resolution image, which was considered as the ground truth.

Tissue probability maps of the template brain derived with SPM and TMBTA priors are provided as both original outputs and smoothed by a Gaussian kernel (FWHM = 0.2 mm).

### 2.5. Template assessment

#### 2.5.1. Template resolution

MRI brain templates, even within the same (sub-)species and imaging modality, often differ in scale, particularly across planes, owing to a range of technical and biological factors introduced during image acquisition. However, their resulting nominal resolution (voxel size) is not sufficient as a quality measure on its own. Other metrics for scan quality are typically only well-defined in artificial settings and phantoms, but do not translate well to *in vivo* imaging and derived templates.

Thus, we employed a parameter-free estimate of microscopy resolution [93] to our data, which has previously been applied to assess the quality of isotropic, super-resolution MRI [94]. In brief, the cross-correlation coefficients *d(r)* are calculated from a normalized, Fourier transformed planar image and its original, with variable filtering (mask radius *r* in Fourier space). The cut-off frequency *k_c_* with the best noise rejection-signal preservation ratio is then used, along with the nominal pixel size, to derive an effective resolution using Equation (1).

(1) $$\text{Resolution}=\frac{2\cdot \text{pixel size}}{k_{c}}$$

This decorrelation analysis was performed for both our template and several preciously published templates. Due to the algorithm assuming square pixels, only planes with isotropic resolution were considered. As contrast and resolvability between adjacent (sub-)structures vary throughout the brain, the overall, minimal calculated effective voxel size was used as criterion for the maximally achieved resolution.

#### 2.5.2. Morphological variability and template differences

To put the FeBls template into perspective, we quantitatively describe the underlying cohort’s and average brain characteristics. Common similarity metrics such as mutual information, cross-correlation, or Dice coefficients provide qualitative measures only, or lack a reference value. They are furthermore sensitive to the images’ different contrast mechanisms and intensities, or only apply to binarized segmentations. Additionally, the optimal (dis-)similarity between templates of different strains, sex, and age is unknown. Thus, all of these similarity measures defeat the purpose and are not reported here. Log Jacobian matrices derived from non-linear registrations between templates, however, may visually indicate spatial similarity and local deformations. Using histograms, one can judge the directionality, magnitude and distribution of spatial transformations.

Transforming one template to another can help locate their differences, but only indicates magnitude and direction, not significance thereof. Thus, we also subjected the axial, anatomical and respective functional baseline scans of 55 mice who fully completed the longitudinal study to the RABIES preprocessing pipeline [53], spatially normalizing their brains. Resulting warp fields were then transformed to log Jacobians and smoothed. In this deformation-based morphology (DBM) analysis, SPM12 was ultimately used to test for regions in our cohort’s brains consistently and significantly being adjusted to match the DSURQE template. Confirming such differences between FeBls and DSURQE template substantiates the independence and necessity of the FeBls brain. As a further check of reproducibility, data of 17 C57Bl/6N males and 33 females recorded a year later with a similar imaging protocol underwent the same procedure.

Lastly, we normalized independent MR data of 42 female C57Bl/6 mice, kindly provided by Martínez-Tazo et al. [32] and Rahman et al. [95], to TMBTA, FeBls, and DSURQE (down-sampled to 60 µm) templates. We then calculated the log Jacobians as a measure of distortion from individual scans to the average space, assuming that the more representative a template of a cohort is, the narrower and more closely centered around zero the log Jacobians are distributed.

## 3. Results

### 3.1. Brain template

Brief characteristics of the imaged cohort and the resulting template can be found in Table 3. The brain template we derived is shown in exemplary coronal, sagittal, and axial slices, as well as a surface rendering in Figure 2. Differences in MR contrast, especially for cerebrospinal fluid, and brain volume compared with the TMBTA brain are well-distinguishable. The former is being attributed to different sequence parameters, such as the higher RARE factor used. The 3D-template itself in NIfTI format and accompanying files have been uploaded to a public repository and are free to download at the Neuroimaging Resources Registry of the NeuroImaging Tools & Resources Collaboratory (NITRC) (see section Data availability). To enhance the applicability of our template, we provided data both in native resolution, and scaled by a factor of 10, for implementation in human-centric analysis software.

**Figure 2.**
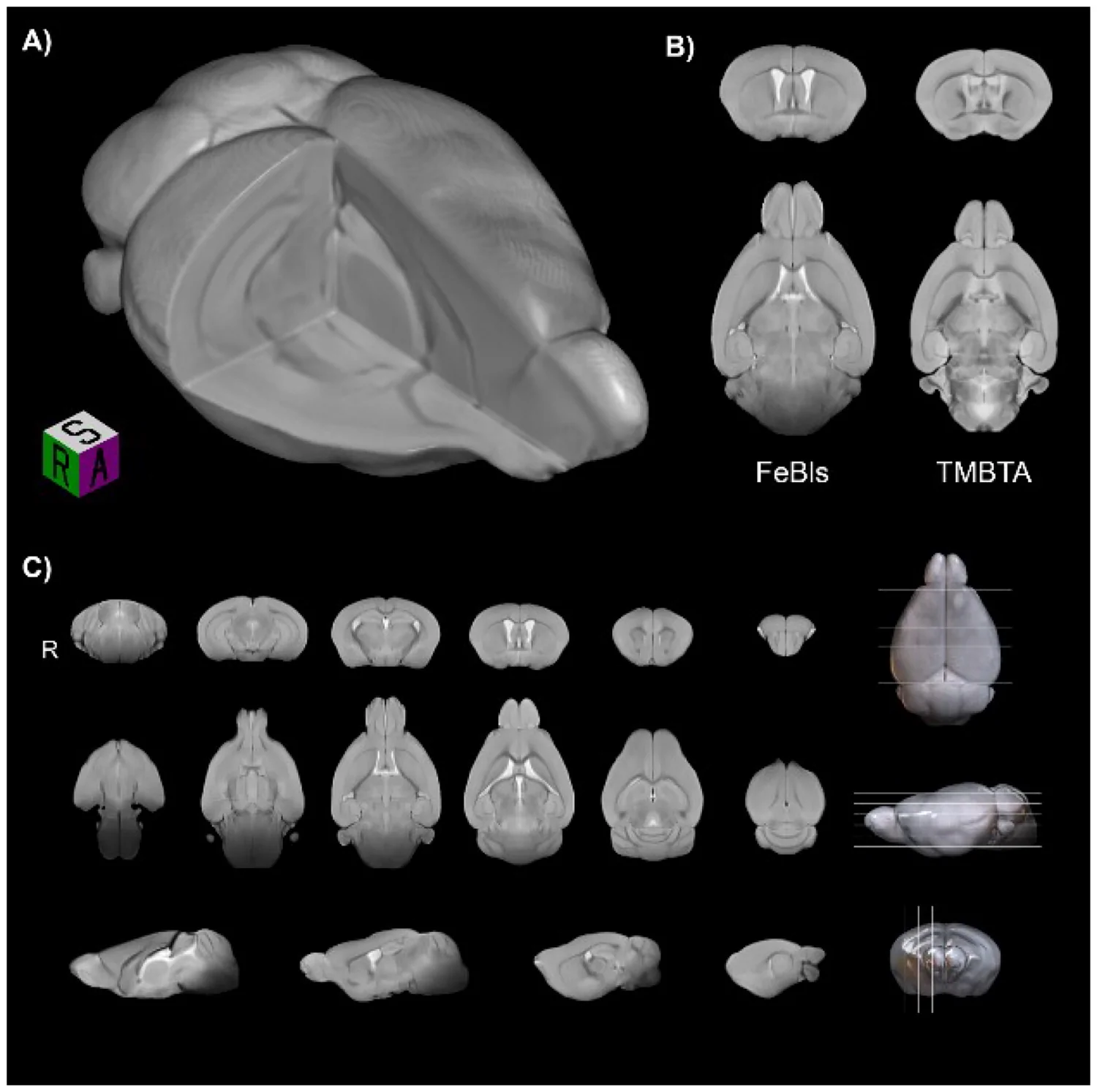
Visualization of the FeBls template with MRIcroGL: **A)** 3D representation with cutout in the right hemisphere, **B)** Visual comparison of size and contrast with the unaltered TMBTA template at a corresponding location and **C)** Mosaic/slice view for the three principal axes.

**Table 3.** Biological and volumetric characteristics of the FeBls template.

N	Weight [g] (mean ± std)	Cohort brain volume [mm^3^] (mean ± std)	Approx. age at arrival [d] (at scan)	Resolution (unscaled)	Template volume [mm^3^]	Scans used of 160 (axial, coronal)
80 ♀ (2 × 40)	22.1 ± 1.9	505.4 ± 18.4	56–76, 70–90 (69–108)	60 µm isotropic	482.6	153 (76, 77)

Figure 3 additionally shows a selection of other templates, spatially warped to FeBls-space (*antsRegistrationSyn.sh -d 3 -m -f -o)*, along with their atlas annotations (*antsApplyTransforms -n GenericLabel*), and FeBls tissue probability maps for gray matter, white matter and cerebrospinal fluid. Due to its importance as a reference for invasive procedures (virus injections, electrode or waveguide placement) and in histological studies, the AMBA was further included as a non-MRI template. With its entirely different resolution and contrast mechanism and limited coverage of the olfactory bulb, the resulting spatial transforms are expected to be less reliable, though.

**Figure 3.**
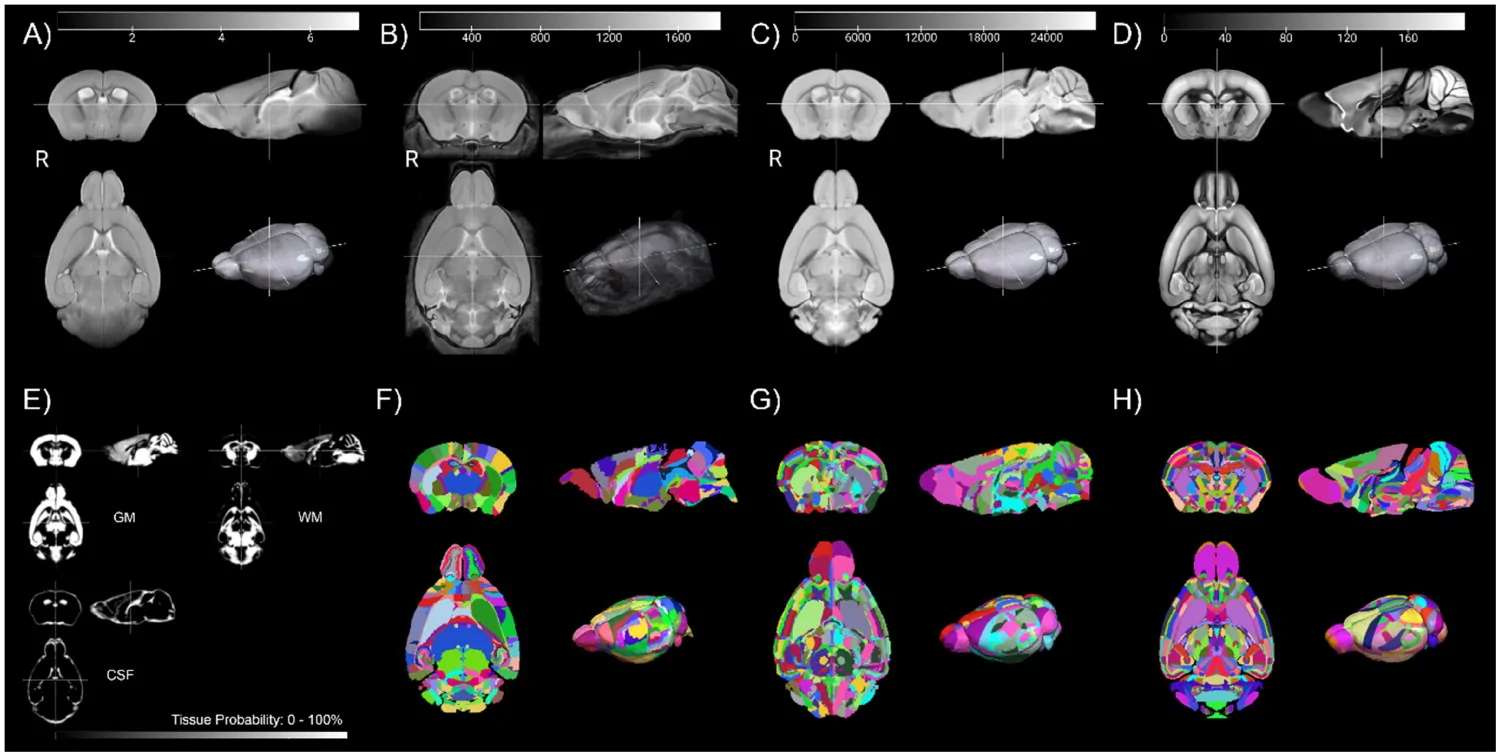
Anatomical annotations and tissue probability maps**:** **A)** FeBls Template Brain **B)** DSURQE template warped to FeBls-space **C)** TMBTA brain warped to FeBls-space **D)** AMBA template warped to FeBls-space **E)** Tissue Probability Maps (clockwise: grey matter (GM), white matter (WM), cerebrospinal fluid (CSF)) **F)** DSURQE anatomical annotations in FeBls-space **G)** TMBTA anatomical annotations in FeBls-space; **H)** AMBA/CCFv3 annotations in FeBls-space. Structural images and respective color bars are in arbitrary units (intensity) and were manually windowed to cover the images’ dynamic range. F-H are for representative purposes only, for details on the annotations, the reader is referred to the data repository and supplementary material.

### 3.2. Effective spatial resolution analysis

We found that the minimal effective voxel size (defined as half the effective resolution, as in [94]) for the isotropic template (constructed from both axial and coronal scans) approached the nominal one (60 µm, < 0.5% difference) for major slice orientations (axial, coronal, sagittal), see also Figure 4. Some rodent templates such as TMBTA [31] and DSURQE [48–52] share this characteristic. A T2w template derived from 10 male C57Bl/6 males by Hikishima et al. [33] has been found to have effective voxel sizes exceeding the nominal resolution in sagittal planes by more than 15%. The Mortimer Space [20] average template’s effective resolution was estimated to be approximately 139 µm, contrasting its nominal resolution of 70 µm. Despite the small variation in effective resolution, a 60 µm isotropic template derived from only axial, non-isotropic scans appeared visually grainier and blurrier on coronal slices. The sagittal and axial slices were perceived to have similar quality, except for the transition of posterior part of the olfactory bulb/the anterior part of the secondary motor area, where the axially derived template exhibited more pronounced borders.

**Figure 4.**
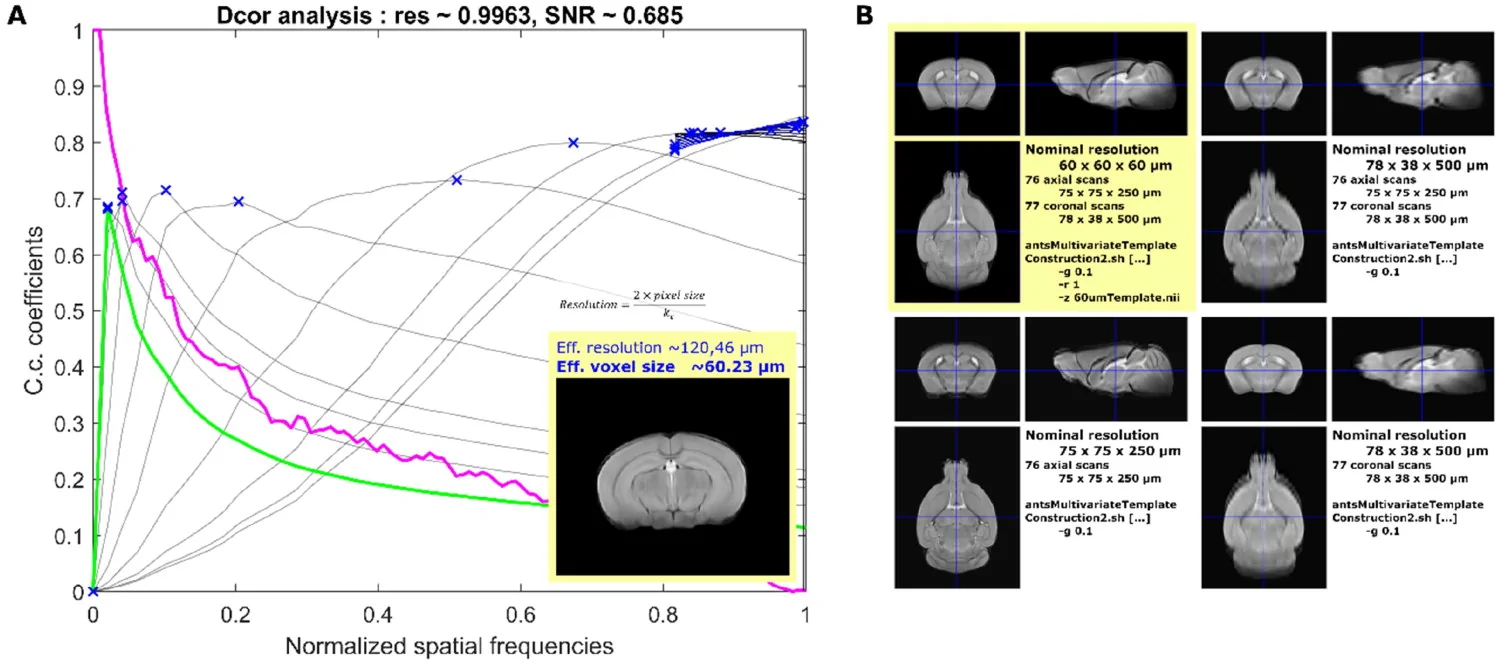
Image resolution analysis **A)** Plot of decorrelation functions used to estimate cut-off frequency $k_{c}$ including the slice analyzed, derived in MATLAB and based on code from [94], see also Code availability. $k_{c}$ is inversely proportional to the effective image resolution. **B)** Templates constructed with different settings and underlying data: Clockwise, starting top left: isotropic template based on two, roughly orthogonal image orientations matched to 60 µm; Anisotropic template based on two scan orientations (Due to no explicit target space being provided, resolution of the template defaults to the first scan provided as input); Templates based on either coronal or axial scans only.

For the single ex-vivo brain scan acquired at high isotropic resolution, we observed that it agreed well, both visually and also regarding anatomical segmentations, with the computationally derived isotropic scan. Furthermore, the parcellated volumes of the computed isotropic scan were on average closer to the ground truth than volumes derived from either axial or coronal scans.

### 3.3. Morphological variability and template differences

In direct comparison, the FeBls template brain (≈ 483 mm^3^) is considerably larger than TMBTA (of significantly younger, RjOrl:SWISS mice, ≈ 346 mm^3^, see also Figure 2(B) and supplement) but differs much less from MouseX_female-only [32] (≈ 495 mm^3^) and DSURQE [48–52] (≈ 487 mm^3^) brain volumes (both C57Bl/6J). Wang et al. further stated that C57Bl/6J total brain volume across sexes averages around 460 mm^3^ at approximately 90 days of age [34], whereas Badea et al. reported more than 508 mm^3^ and standard deviation of ≈ 23.42 mm^3^ for a cohort of 9 weeks old C57Bl/6J mice [96]. This closely matches our cohort’s average of 505 ± 18 mm^3^ (averages within subjects across parcellations of either slice orientation or resolution).

Looking at the TMBTA atlas segmentations specifically, which encompasses the same regions at the same nominal resolution, as well as the warp fields from one template to the other and the resulting log Jacobians (Figure 6), it becomes apparent that the volume difference is not merely caused by overall scaling of the whole brain, but that different brain regions scale to a different extent. Despite the lower total volume (resulting in negative log Jacobians, as the FeBls template needs to shrink to match the TMBTA, Figure 6(A)), some small brain regions may be larger in the TMBTA than in the FeBls template. However, as these are generally located closer to the trunk, this effect could be influenced by the different contrast in our acquisitions and lower signal intensity. In such low-contrast regions, reduced segmentation performance is to be expected. The DSURQE template is not only more similar in overall volume and dimensions, but also the log Jacobian values are, on average closer to zero.

Visually, the overall shape of the FeBls brain appears less round along the anterior-posterior axis, when drawing a straight line between the base of the olfactory bulb and proximal-superior part of the midbrain, but extends slightly more downwards at the height of hypothalamus and medial septum (Figure 6, top left). Among other regions, pronounced differences between DSURQE and FeBls were observed for parts of the olfactory bulb, nucleus accumbens, hippocampus, thalamus and bed nuclei of the stria terminalis (Figure 5(A)). As shown with DBM (Figure 6), regions significantly different between DSURQE and FeBls’ underlying data are the thalamus, hippocampus, insula and piriform cortex, many of which are known to be either dimorphic or involved in differently expressed behavioral phenotypes between C57Bl/6N and 6J. Findings in hippocampus (dentate Gyrus, CA3) and corpus callosum were particularly robust and consistent in C57Bl/6N females and males. Sex differences found in an exploratory analysis (e.g. cerebellum, mesencephalon, primary somatosensory cortex, hippocampus and lateral septum) should be verified in a more balanced sample.

**Figure 5.**
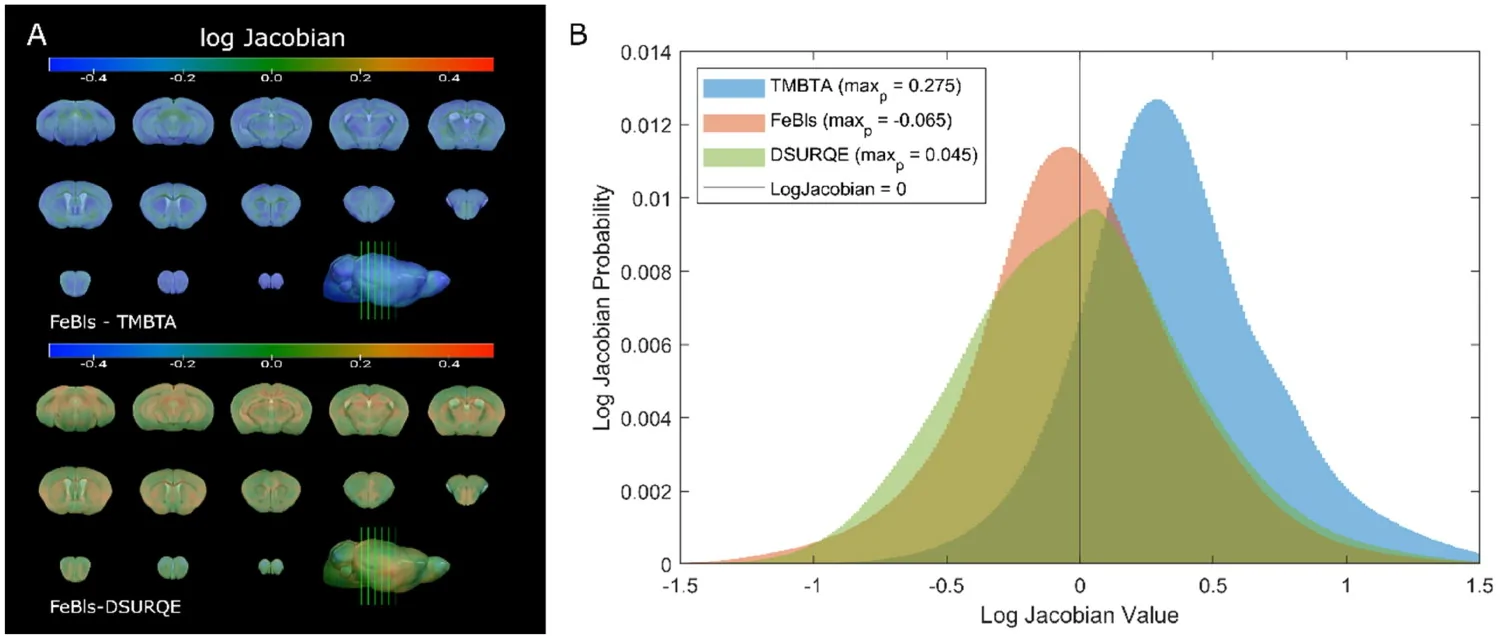
Spatial transformations between and towards brain templates **A)** Log Jacobian maps for spatial transformations between FeBls and TMBTA space (top) and FeBls and DSURQE templates (bottom) **B)** Distributions of log Jacobian values when normalizing external data of female mice to TMBTA, FeBls and DSURQE templates, respectively.

Consistent with aforementioned observations in the template cohort, externally scanned, adult female C57Bl/6 brains were on average larger than TMBTA, as seen by the shift towards the right in the distribution of log Jacobian values (Figure 5(B)). As hypothesized, for the three templates at the same resolution, the distribution corresponding to normalization to FeBls is both narrow and most symmetric around zero (Figure 5(B)), indicating a good agreement between the independent data and the template.

**Figure 6.**
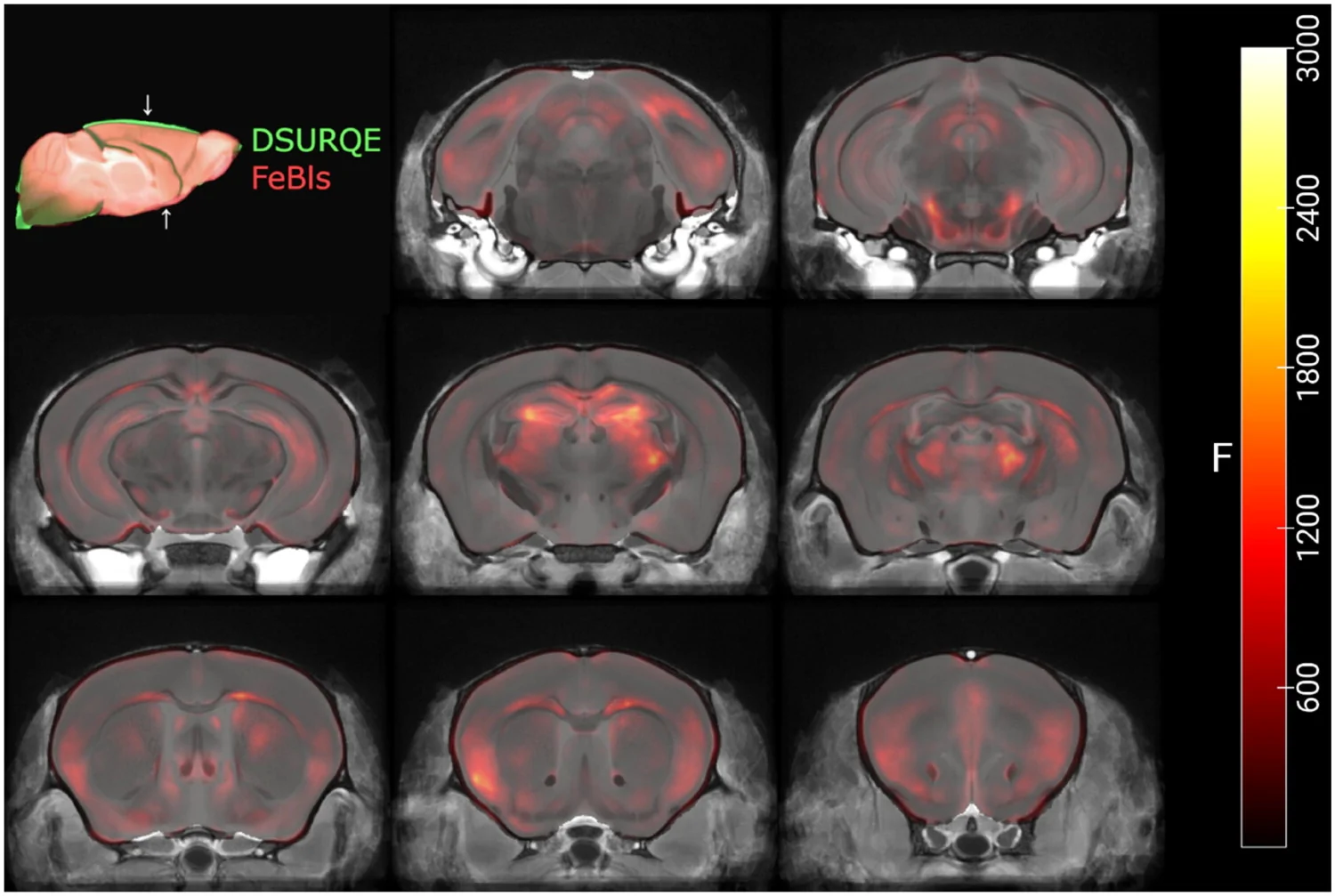
Significant differences between DSURQE and FeBls template. Top left: Templates were manually aligned and linearly scaled to display overall shape differences. Arrows indicate regions of reduced overlap. Axial slices of the DSURQE template are shown with a statistical map, indicating significant deformations when spatially normalizing C57Bl/6N female brains towards DSURQE space (F = [36.32, 3000], p_FWE_ < 0.05).

## 4. Conclusions

Herein, we present a novel brain template, aiming to address the paucity of female mouse template brains and C57Bl/6N strain, specifically. We show that the FeBls template significantly differs from mixed-sex and female-only (C57Bl/6J) references. The evaluation of external data not used for generation of the FeBls template showed a narrower and more symmetric log Jacobian distribution than with DSURQE, indicating a closer match between female individuals and our reference.

Although we lack a direct comparison, given partially consistent structural findings in three data sets (55 and 33 females, 17 males), we have reason to assume that some, but not all differences in templates are caused by mixed-sex versus female-only data. We hypothesize that in a sufficiently large sample also C57Bl/6N and C57Bl/6J are anatomically distinct, and template differences between FeBls and DSURQE shown are partially strain-specific. Whether the rate of congenital anomalies and variability (portosystemic shunt) drive these differences, and their respective prevalence in C57Bl/6N are still to be determined in future studies.

### 4.1. Limitations

Naturally, our work is not without limitations: Despite best efforts, transformations between templates and resulting annotations may exhibit local deviations. Thus, atlas files should be used cautiously and placement of volumes of interest should be individually verified. A general issue with anatomical atlases is their varying degree of parcellations and usage of inconsistent nomenclature. Unifying and implementing levels of anatomical hierarchy (e.g. in 4D annotation files) is important and should be covered in future work.

While in many mouse studies narrow age ranges are used, we explicitly opted for a wider range to truly reflect adult brain morphology and broaden applicability. However, this may not fully align with study designs where the earlier sexual, instead of the brain morphological maturity is considered as criterion for adulthood.

Finally, covering a broader range of (structural) imaging contrasts (T1-weighted, proton density, diffusion) was unfortunately not possible within the experimental timeline and scope of this study.

## Supplementary Material

Supplemental Material

SUPPLEMENTARY FIGURE 1.docx

## Figures and Tables

**Table:** 

*antsMultivariateTemplateConstruction2.sh*	
*-d 3*	Input image dimensions i.e. 3D, compulsory
*-r 1*	Rigid-body registration of inputs to initial template (space)
*-z templateSpace.nii*	Initial target volume (space) for inputs, final template will have same resolution
*-g 0.1*	Gradient step size
*-v 250*	Memory limit
*-c 2*	Parallel computation
*-j 15*	Number of CPUs
*-n 0*	N4BiasFieldCorrection: Off (as previously applied on input data)
/folder/*.nii	List of input files specified by e.g. folder and file extension

## Data Availability

As previously stated, the following zipped files are freely available at two scales (native and upscaled ‘10x’, if applicable) as downloads at https://www.nitrc.org/projects/febls (current version: 1.4): FeBls Template Brain FeBls Anatomical Annotations (TMBTA, DSURQE, AMBA, MouseX female, Dorr) FeBls TPM FeBls Brain Mask Spatial transformations between template spaces Raw data and individual scans will be made available upon request.

## Notes

1 Given the focus of this paper on animal experimental neuroscience research, we herein refer to sex solely as a binary, biological trait determined by a set of chromosomes. “Female” is thus used for individuals carrying exactly two X-chromosomes (40,XX in mice), which typically result in female reproductive organs (which in mice would commonly be used as the single classifier for sex at weaning). Nevertheless, we acknowledge human sex and gender (identity) as separate and more complex entities that do not necessarily converge and which may shape brain structure and function differently.

2
https://civmimagespace.civm.duhs.duke.edu

3
https://scalablebrainatlas.incf.org/mouse/ABA_v3

4
https://www.mouseimaging.ca/DSURQE_40micron.tar.gz

5 http://www.bmap.ucla.edu/portfolio/atlases/Mortimer_Space_Atlas/.

## Code availability statement

Templates were constructed using the ANTs software package. An exemplary call to the respective function may look like the one below (arguments that should not influence the outcome and depend on hardware available in gray):

For further details, the reader is referred to the software manual or may call respective functions with ‘-h’ as argument.

The effective image resolution was estimated in MATLAB for single MR-slices by calling the function

[kcMax,A0,kcGM,d0,d] = getDcorr(im,r,Ng,figID)

provided here: https://github.com/Ades91/ImDecorr.git. The effective voxel size was then estimated by dividing the nominal voxel size (as given in the image header) by kcMax. Analogous to [94], the factor 2 was omitted, which describes the minimal distance required between two points to be distinguishable in an image.

Further software is freely available here (GNU Octave may be used alternatively instead of MATLAB (not tested))

https://github.com/ANTsX/ANTs

https://github.com/spm

https://download.slicer.org/
