# Supplementary material for: An MRI template of the female C57Bl/6N mouse brain
Source: Ann Med. 2026 Nov 11;58(1):2753239. doi: 10.1080/07853890.2026.2753239 (PMC13761078; doi:10.1080/07853890.2026.2753239)
Supplement: Supplementary file 2 [file IANN_A_2753239_SM5404.docx]

**SUPPLEMENTARY FIGURE 1**

Supp. Fig. 1 3D-printed phantom The phantom (rendered computer model, center) was printed in resin, featuring a live-size mouse skull (Mouse Skull and Brain, by smcinturff 2018, https://www.thingiverse.com/thing:3079327) attached to an orthogonal grid pattern. The phantom was scanned on a CT (bottom), placed in a 15 ml Falcon tube and imaged on the MR scanner (top). Due to the CryoCoil’s small field of view, only the ends of the phantom could be measured. The resin showed little radiological contrast (Hounsfield units) compared to water, thus was scanned on its own in the CT. No significant distortion and geometrical differences in co-registered image modalities were discerned, except for local effects of small air-bubbles in water.
